# Enhancing the Cosmetic Potential of Aloe Vera Gel by Kombucha-Mediated Fermentation: Phytochemical Analysis and Evaluation of Antioxidant, Anti-Aging and Moisturizing Properties

**DOI:** 10.3390/molecules30153192

**Published:** 2025-07-30

**Authors:** Aleksandra Ziemlewska, Martyna Zagórska-Dziok, Anna Nowak, Anna Muzykiewicz-Szymańska, Magdalena Wójciak, Ireneusz Sowa, Dariusz Szczepanek, Zofia Nizioł-Łukaszewska

**Affiliations:** 1 Department of Technology of Cosmetic and Pharmaceutical Products, Medical College, University of Information Technology and Management in Rzeszow, Sucharskiego 2, 35-225 Rzeszow, Poland; aziemlewska@wsiz.edu.pl (A.Z.); mzagorska@wsiz.edu.pl (M.Z.-D.); 2 Department of Cosmetic and Pharmaceutical Chemistry, Pomeranian Medical University in Szczecin, 72 Powstańców Wielkopolskich Street, 70-111 Szczecin, Poland; anna.nowak@pum.edu.pl (A.N.); anna.muzykiewicz@pum.edu.pl (A.M.-S.); 3 Department of Analytical Chemistry, Medical University of Lublin, Aleje Raclawickie 1, 20-059 Lublin, Poland; magdalena.wojciak@umlub.pl (M.W.); ireneusz.sowa@umlub.pl (I.S.); 4 Department of Neurosurgery and Paediatric Neurosurgery, Medical University of Lublin, 20-090 Lublin, Poland; dariusz.szczepanek@umlub.pl

**Keywords:** aloe vera gel, kombucha ferments, cosmetic properties, collagenase, elastase, hyaluronidase activity, skin cells, skin permeability, antioxidants, moisturizing activity

## Abstract

Aloe vera gel is a valuable raw material used in the cosmetic industry for its skin care properties. The present study analyzed the effects of the fermentation of aloe vera gel with a tea fungus kombucha, which is a symbiotic consortium of bacteria and yeast, carried out for 10 and 20 days (samples F10 and F20, respectively). The resulting ferments and unfermented gel were subjected to chromatographic analysis to determine the content of biologically active compounds. The permeability and accumulation of these compounds in pig skin were evaluated. In addition, the methods of DPPH, ABTS and the determination of intracellular free radical levels in keratinocytes (HaCaT) and fibroblasts (HDF) cell lines were used to determine antioxidant potential. The results showed a higher content of phenolic acids and flavonoids and better antioxidant properties of the ferments, especially after 20 days of fermentation. Cytotoxicity tests against HaCaT and HDF cells confirmed the absence of toxic effects; moreover, samples at the concentrations tested (mainly 10 and 25 mg/mL) showed cytoprotective effects. The analysis of enzymatic activity (collagenase, elastase and hyaluronidase) by the ELISA technique showed higher levels of inhibition for F10 and F20. The kombucha ferments also exhibited better moisturizing properties and lower levels of transepidermal water loss (TEWL), confirming their cosmetic potential.

## 1. Introduction

*Aloe barbadensis Miller* (aloe vera) is a tropical plant of the *Liliaceae* family that has been valued in the traditional medicine of various cultures for centuries. The gel extracted from the thin-walled parenchymatous cells of the leaves exhibits a broad spectrum of biological activity, which is reflected in its applications in the food, pharmaceutical and cosmetic industries [1,2]. Aloe vera has been extensively studied for its cosmetic properties, particularly due to its rich composition of bioactive compounds such as vitamins, enzymes, polysaccharides and amino acids [3,4]. These components contribute to its moisturizing, anti-inflammatory, antimicrobial and healing effects, making it a valuable ingredient in skin care formulations [5,6]. Among the components of particular clinical interest is acemannan, a polysaccharide responsible for stimulating the immune response and accelerating fibroblast proliferation, making it an important factor in promoting wound healing and epithelial reconstruction. In addition to its immunostimulating effect, acemannan exhibits antiviral and inflammation-relieving properties [7,8,9]. Aloesin, a chromone with the ability to inhibit melanin synthesis by reducing tyrosinase activity, is also used in cosmetological practice, giving it potential in the treatment of skin discoloration and pigmentation disorders [10]. In addition, the presence of phytosterols (e.g., lupeol and campesterol) and plant hormones (auxins and gibberellins) as well as biologically active compounds such as emodin and aloin, which enhance anti-inflammatory effects, promote tissue healing and the regulation of metabolic processes [11].

The fermentation of plant raw materials is increasingly used in modern cosmetology as a method of intensifying the action of active ingredients. One example of such raw materials is kombucha ferment, which is the product of fermenting sweetened tea by a symbiotic culture of bacteria and yeast known as SCOBY (*Symbiotic Culture of Bacteria and Yeast*). This process leads to the formation of a mixture of biologically active compounds that can exert beneficial effects on skin condition [12]. Unlike classical ferments used in cosmetics, such as aloe vera ferments with lactic acid bacteria (mainly of the genus *Lactobacillus* and *Bifidobacterium*) or yeast (*S. cerevisiae*), kombucha ferment is characterized by a different metabolic profile [13,14,15]. It includes glucuronic acid, acetic acid, B vitamins, polyphenols and enzymes, among others, which are the result of the interaction of microorganisms present in the SCOBY culture. These compounds exhibit antioxidant, anti-inflammatory, brightening and skin regenerative properties [12,16]. Unlike plant extracts, fermentation using kombucha not only increases the bioavailability of active compounds, but also creates new metabolites with biological potential that are not present in the unfermented starting material. Thus, kombucha ferment represents an interesting and innovative alternative for the extraction of bioactive ingredients for modern cosmetic products [17].

The aim of this study was to compare the content of biologically active compounds and biological properties in Aloe vera gel and its ferments obtained after 10 and 20 days of fermentation with kombucha tea fungus. As part of the study, key active compounds were determined using liquid chromatography coupled with the mass spectrometry (LC-MS) technique. Given the key role of oxidative stress in skin aging and disease, antioxidant activity was also assessed using DPPH and ABTS assays, and intracellular free radical levels were measured in keratinocyte (HaCaT) and human skin fibroblast (HDF) cell lines. Reactive oxygen species (ROS) damage lipids, proteins and DNA, contributing to inflammation, collagen breakdown and skin dysfunction [18,19,20]. ROS are also associated with hyperpigmentation, barrier disruption and chronic skin diseases such as atopic dermatitis and psoriasis [19,20,21,22]. Assessing the antioxidant capacity of fermented and unfermented Aloe vera therefore highlights their protective potential against oxidative stress. Cytotoxicity against these cells was determined using Alamar Blue and Neutral Red assays. Given the widespread use of Aloe vera in cosmetics, special attention was also paid to the analysis of anti-aging properties by determining the activity of enzymes responsible for the degradation of the extracellular matrix, i.e., collagenase and elastase as well as ashyaluronidase, using the ELISA technique. An essential factor in cosmetic development is the ability of active ingredients to penetrate the skin barrier and exert effects in deeper layers. The stratum corneum (SC), rich in lipids, presents a major obstacle to this process. Penetration depends on compound properties such as polarity, lipophilicity and molecular size [23,24,25,26,27,28,29], making permeation studies crucial for evaluating dermal bioavailability. In the context of the moisturizing properties of Aloe vera gel, the permeability of active compounds through pig skin was tested and quantified. In addition, corneometric and tewametric measurements were used to evaluate the effect of hydration and transepidermal water loss (TEWL).

## 2. Results and Discussion

### 2.1. Determination of Bioactive Compounds

Anthrones, primary aloin and its derivatives, and chromone derivatives were the main components found in the investigated Aloe extract. A common feature of aloin derivatives is the characteristic three UV absorption maxima in the regions of 260–270 nm, 290–300 nm and 355–360 nm. Meanwhile, chromones show maximum absorption in the regions of 215–225 nm, 245–255 nm and 295–305 nm. Further identification included MS analysis in both negative and positive ionization modes. Typical base peak chromatograms are shown in Figure S1. Compounds were identified based on mass data, UV spectra, elution order and comparison with literature data (Table S1). The UV and mass spectra of the predominant compounds identified as aloin (*m*/*z*-H = 417) and aloesin (*m*/*z*-H = 393) are presented in Figure S2.

**Figure 1:**
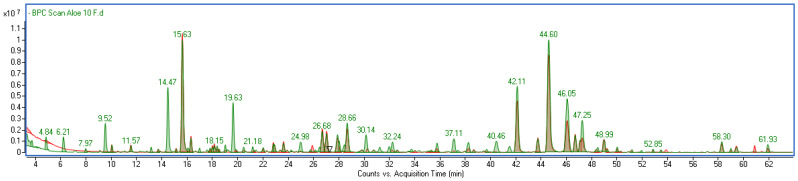
Overlapped base peak chromatograms of Aloe vera gel (red) and its ferments (green).

The comparison between the unfermented Aloe extract (AG) and the fermented samples (F10 and F20) revealed significant compositional changes resulting from the fermentation process (Figure 1). Fermentation led to the appearance of several additional peaks on the chromatograms that were absent in the AG sample (Figure 1). Although some of these compounds were also detected in the kombucha alone, they were present only in trace amounts (Figure S3). The quantitative analysis of the extracts showed a general trend of increasing polyphenolic content with longer fermentation time. The most pronounced increases were observed for gallic acid (c.a. 78%), galloylquinic acids (33%), (epi)gallocatechins (30%), chlorogenic acids (37%), epicatechin (45%) and flavonoids in total (60%) when comparing 10 and 20 days of fermentation. This suggests that fermentation promotes the release or formation of these bioactive compounds. The detailed results of quantification are summarized in Table 1.

The observed increase in flavonoid and phenolic compound concentrations following fermentation is primarily attributed to the enzymatic activity of the kombucha microbial consortium, which includes bacteria and yeasts capable of producing enzymes such as β-glucosidases, esterases and tannin acyl hydrolases. These enzymes may hydrolyze high molecular polymeric forms naturally present in the extract into smaller molecules what was reported in some papers [30,31]. In addition, kombucha microbes can perform biotransformations through metabolic pathways such as deglycosylation, ring fission, methylation and dihydroxylation. These transformations may lead to the formation of new metabolites not originally present in the unfermented Aloe extract, including flavonoid derivatives and phenolic acids, thereby enriching the phytochemical profile of the fermented product. Such microbial bioconversion during kombucha fermentation has previously been reported in the literature [32,33,34].

### 2.2. Penetration Study

The experiment was conducted using Franz diffusion cells and pig skin. Due to the limited availability of human skin and the associated ethical issues, porcine skin is commonly used as a preliminary model for the assessment of the transdermal transport of active substances. Numerous histopathological studies have shown its similarity to human skin both in terms of the thickness of the epidermal layers (basal, spinous and granular) and the number of cells in the stratum corneum. The lipid composition of the SC of human and porcine skin is also very similar, which makes this model reliable [35].

**Figure 2:**
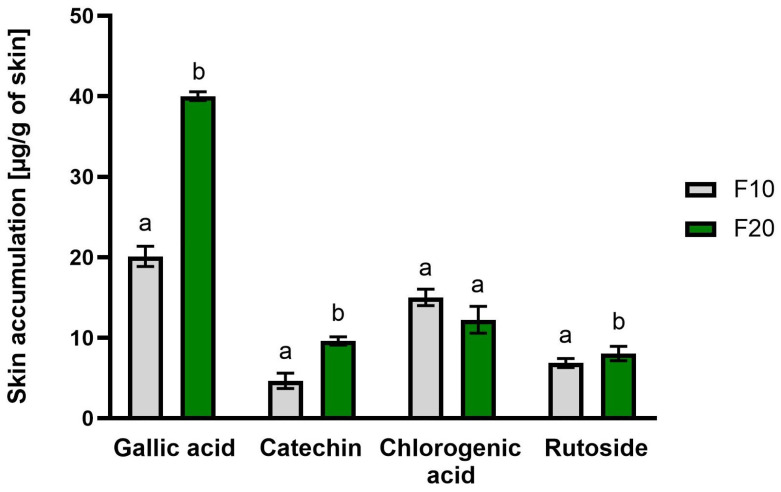
Accumulation in the skin of selected active substances after 24-h study, n = 3; different letters indicate significant differences between the tested extracts; α = 0.05.

The skin permeation of selected active substances from 10-day (F10) and 20-day ferments (F20) through the skin of pigs during a 24-h study using a Franz diffusion cell is presented in Table 2. The accumulations of selected compounds in the skin are presented in Figure 2. Penetration studies were not performed on the aqueous extract of Aloe vera (AG) because no phenolic acids or flavonoids relevant for skin action were identified in it. The study was conducted for the following active compounds: gallic acid, chlorogenic acid, catechin and rutin. All analyzed secondary metabolites penetrated the skin and accumulated in it. In the case of gallic acid, it was observed that it permeated through the skin to the acceptor fluid only in the fifth hour after application to the skin for both ferments. The concentration of this compound after 24 h of the study was similar for both ferments and amounted to 14.42 ± 2.69 and 14.05 ± 2.69 μg/cm^2^, respectively, for F10 and F20. In the case of catechin, a significantly greater amount was observed in the acceptor fluid for F10 after 24 h of analysis (9.41 ± 1.94 μg/cm^2^). Similarly, ruzoside permeated in a significantly greater amount; in the case of F10, it permeated by the fifth hour of the study, while in the case of F20, it did not permeate until the 24th hour (Table 2). In our study, we observed a generally lower penetration of secondary metabolites for the gel subjected to longer fermentation (F10). Some strains of bacteria present during the fermentation of kombucha mushroom (SCOBY) can produce exopolysaccharides (EPS), which are specific biopolymers including dextran, levan, glucan, gellan and cellulose, among others. Such extracellular biopolymers are responsible for contributing to the specific characteristics of fermented products, including modifying their organoleptic properties. Therefore, they can change the structure and viscosity of the gel [36], which, in our study, could be the reason for the significantly lower penetration of secondary metabolites after a longer period of fermentation of Aloe vera gel. All tested compounds also accumulated in the skin. The highest amount was accumulated by gallic acid, which in the F10 sample accumulated in the amount of 40.00 ± 0.55 μg/g skin after application (Figure 2).

### 2.3. Assessment of Antioxidant Activity

#### 2.3.1. DPPH and ABTS Radical Scavenging

In the conducted studies, the antioxidant activity of Aloe gels and ferments was assessed using the DPPH and ABTS methods. Both methods are related to the reaction of the DPPH and ABTS radicals with electron donors or hydrogen atoms, which results in a change in the color of the tested solution. The gels and bioferments that were fermented for 10 and 20 days, respectively, were analyzed. The samples were tested at concentrations of 5, 10, 25 and 50 mg/mL.

**Figure 3:**
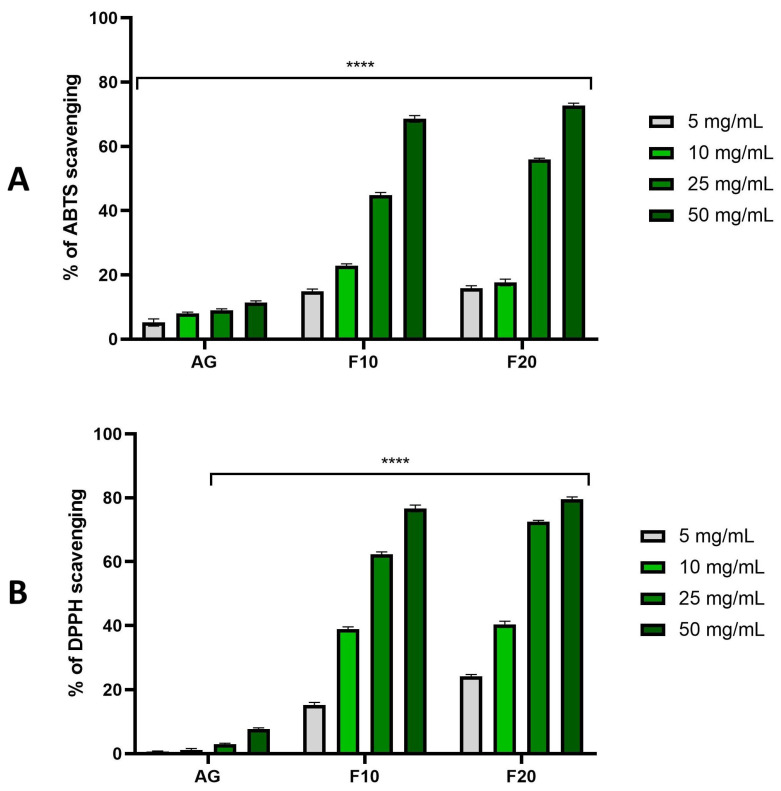
The ability of Aloe vera gel (AG) and ferments (F10 and F20) to scavenge ABTS (**A**) and DPPH (**B**) free radicals at concentrations of 5, 10, 25 and 50 mg/mL. Data are presented as mean ± SD from three independent experiments, with each sample tested in triplicate. **** *p* < 0.0001.

The results obtained in the experiments performed with DPPH and ABTS were very close, confirming each other. The following observations were made. The first is that, in each series of experiments, there was a clear correlation between concentration and antioxidant ability, making the observed values higher with the higher concentration of a sample. This held true both for pure extracts as well as for ferments. Another observation was that both ferment variants of F10 and F20 exhibited much stronger antioxidant abilities compared to the pure non-fermented extracts, where the difference was very high across all the concentrations, especially for concentrations of 50 mg/mL. The observed values were close to 80% for DPPH and around 70% for ABTS, while for pure extracts, all of the values remained around or just slightly over 10%. Another observation was that longer fermentation resulted in visibly higher antioxidant properties in higher tested concentrations such as as 25 and 50 mg/mL. To summarize, after the first 10 days of fermentation a very significant jump of antioxidant properties was observed, while after another 10 days, registered values were even higher, but the increase compared to the 10-day ferments was rather moderate (Figure 3).

The antioxidant properties of Aloe vera have been previously documented by several authors using DPPH and ABTS assays. Notably, most of these studies have concentrated on characterizing the radical scavenging activity of different types of Aloe extracts or powdered preparations. Hossen et al. and Mishra et al., working within distinct research groups, independently reported that both the method of sample dehydration and the choice of extraction solvent exert a significant influence on the in vitro antioxidant capacity of Aloe-based preparations, underscoring the critical role of processing parameters in modulating bioactivity [37,38]. Similarly, Ray and co-authors highlighted the importance of the plant’s growth stage, showing that the antioxidant capacity against DPPH radicals varies considerably depending on the harvest time of the Aloe material [39]. In another study, Wintola and Afolayan evaluated extracts obtained from powdered dried Aloe leaves and confirmed their free radical scavenging capabilities. Their findings revealed that the extracts exhibited greater efficacy against ABTS radicals compared to DPPH, pointing to differential reactivity depending on the assay system used [40].

In the unfermented Aloe gel, a slightly higher ABTS scavenging activity was observed in comparison to DPPH, particularly at lower concentrations. This outcome may be linked to the hydrophilic nature of the native matrix, which is rich in polysaccharides, phenolic acids and other water-soluble antioxidants, all of which are known to interact more effectively with ABTS radicals. Since ABTS is soluble in both aqueous and organic media and reacts via both hydrogen atom transfer (HAT) and single electron transfer (SET) mechanisms, it is particularly suitable for evaluating antioxidant potential in hydrophilic systems [41]. In contrast, both F10 and F20 ferments exhibited a notable shift in scavenging capacity, with significantly greater activity toward DPPH radicals. This inversion may be attributed to biochemical transformations during fermentation, which involve the microbial degradation of high-molecular-weight polysaccharides and the release or biosynthesis of low-polarity phenolic compounds, aglycones and secondary metabolites. These fermentation-derived compounds tend to have higher affinity toward the DPPH radical, which is relatively lipophilic and preferentially reacts via HAT mechanisms [42,43,44]. Therefore, the enhanced DPPH scavenging following fermentation was likely a consequence of increased concentrations of lipophilic or semi-lipophilic antioxidants, which were either liberated from glycoside forms or newly formed through microbial metabolism. Overall, fermentation not only amplified the antioxidant potential of aloe-based preparations but also altered the radical scavenging profile, shifting the balance toward a greater reactivity with DPPH radicals, possibly due to structural and polarity changes in the antioxidant pool [45,46,47].

#### 2.3.2. Intracellular ROS Levels in Skin Cells

In addition, the ability to generate intracellular production of reactive oxygen species was assessed on the cell lines fibroblasts (HDF) and keratinocytes (HaCaT). These analyses were performed using the fluorogenic dye H_2_DCFDA, which is oxidized in the presence of reactive oxygen species and converted into highly fluorescent 2′,7′-dichlorofluorescein (DCF). As a positive control, 500 µM hydrogen peroxide (H_2_O_2_) was used, which was used to treat the analyzed cell lines. The negative control was cells not treated with the test compounds and hydrogen peroxide.

**Figure 4:**
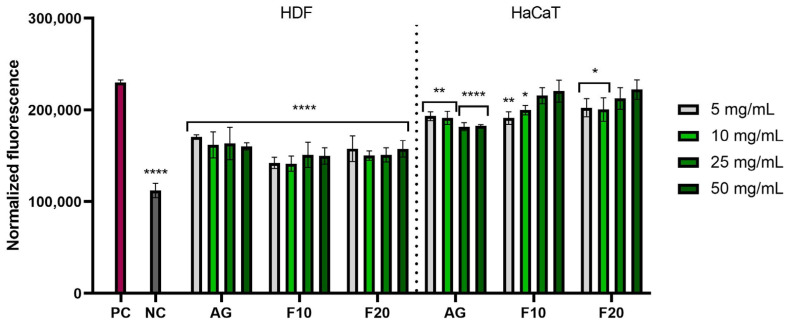
The effect of Aloe vera gel (AG) and ferments (F10 and F20) at the concentrations of 5, 10, 25 and 50 mg/mL on the intracellular level of reactive oxygen species in fibroblasts (HDFs) and keratinocytes (HaCaT). Data are presented as mean ± SD from three independent experiments, with each sample tested in triplicate. **** *p* < 0.0001, ** *p* < 0.01, * *p* < 0.05.

The analyses carried out showed that all types of extracts used in the experiment had the ability to reduce oxidative stress in both ROS HDF and ROS HaCaT. In both ROS HaCaT and ROS HDF experiments, there was no clear correlation between the concentration of the sample and the values observed, meaning that increasing the concentration of extracts did not cause a visible increase in the ability to reduce oxidative stress. Depending on the case within a given sample, observed values remained on average on similar level, sometimes slightly increasing with concentration, increasing in one particular concentration, or even fluctuating up and down when the concentration was increased. Despite a lack of clear correlation with the concentration, the results obtained for all the samples remained at a lower level compared to positive control, indicating antioxidant properties. Lower values, and thus a higher ability to reduce intracellular free radicals, were observed for HDF cells, which obtained statistically significant results for all tested concentrations. For HaCaT cells, Aloe vera gel (AG) at concentrations of 25 and 50 mg/mL showed the most favorable effects (Figure 4).

Numerous scientific studies indicate that secondary metabolites of Aloe plants can effectively support the protection of cells against oxidative stress, contributing to the increased activity of antioxidant enzymes, reducing lipid peroxidation levels and delaying skin aging processes. In addition, the antioxidant properties of Aloe vera provide a protective effect, accelerate regeneration and eliminate skin discoloration [19,21,48,49]. Also, in the case of the conducted studies of Aloe gel and its ferments, a significant ability to neutralize free radicals was demonstrated, which is closely related to the content of polyphenolic compounds such as chlorogenic, cinnamic or ferulic acid; moreover, it corresponds to the content of flavonoids or anthraquinones such as aloesin and aloin. The conducted analyses showed the presence of the above-mentioned compounds in the analyzed samples, which is related to the strong antioxidant properties of the analyzed material. Moreover, the results clearly showed that extracts fermented with kombucha were characterized by stronger antioxidant activity compared to unfermented extracts, which corresponded with the results regarding the content of compounds with antioxidant potential.

### 2.4. In Vitro Assessment of Cytotoxicity on Skin Cells

**Figure 5:**
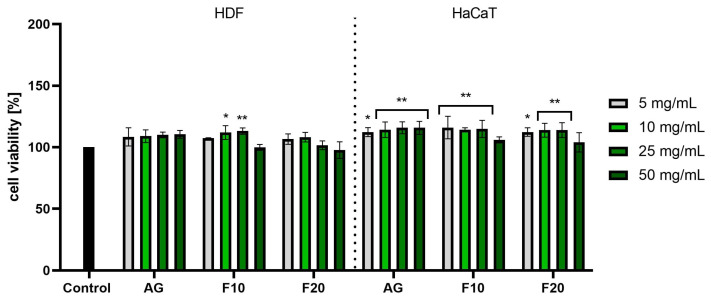
Assessment of resazurin reduction in fibroblasts (HDF) and keratinocytes (HaCaT) by Aloe vera gel and ferments in the concentration range of 5–50 mg/mL. The study used a gel (AG) and a 10-day (F10) and 20-day ferment (F20) obtained after fermentation of the gel with kombucha. Control cells were cells maintained in the medium without the addition of the tested samples, for which the viability was assumed to be 100%. ** *p* < 0.01, * *p* < 0.05.

The cytotoxicity of Aloe gel and its ferments was assessed using the Alamar blue (AB) and Neutral red (NR) assays. This allowed for the assessment of both the metabolic activity of the tested cells and the quantitative measurement of cell viability by measuring the amount of uptake of supravital dye (neutral red) in the lysosomes of living cells. The analyses were carried out on both dermal cells (HDF fibroblasts) and epidermal cells (HaCaT keratinocytes). In the case of the first test used to measure the reduction of resazurin, no cytotoxic effect was observed for any of the concentrations used, both for Aloe gel and ferments. The lack of cytotoxicity of the tested samples (in the tested concentration range) was confirmed on both tested cell lines. In the case of HDF cells, the 10 and 25 mg/mL concentrations of F10 caused a statistically significant increase in the viability of these cells: up to 111.92 ± 5.57% and 113.08 ± 2.57%, respectively. In the case of HaCaT cells, all of the tested concentrations of gel and its ferments showed a positive effect on the viability of these cells, reaching a maximum viability of about 115% for AG and F10 at the three lowest concentrations tested (Figure 5).

**Figure 6:**
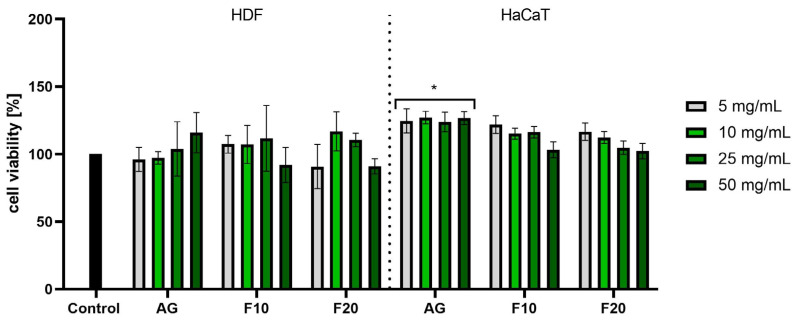
Assessment of neutral red uptake in fibroblasts (HDF) and keratinocytes (HaCaT) by Aloe vera gel and ferments in the concentration range of 5–50 mg/mL. The study used a gel (AG) and a 10-day (F10) and 20-day ferment (F20) obtained after fermentation of the gel with kombucha. Control cells were cells maintained in the medium without the addition of the tested samples, for which the viability was assumed to be 100%. * *p* < 0.05.

In the case of the NR assay, much greater differences were observed between the effects of Aloe gel and its ferments, especially in the case of HDF cells. Analyses performed on fibroblasts did not show a statistically significant effect of the gel on the viability of these cells; however, in the case of the gel obtained after 10 days of fermentation used at a concentration of 50 mg/mL, a slight decrease in the viability of these cells to 92% was observed. In the case of keratinocytes, a slight increase in their viability was observed after the use of all tested concentrations of gel. However, in the case of F10 and F20, no statistically significant effects of these samples on HaCaT cell viability were observed at any of the tested concentrations. The lower concentrations used affected the proliferation of the cells tested (Figure 6).

To strengthen the interpretation of the observed effects and address the potential limitations associated with the use of metabolic assays such as resazurin (Alamar blue assay), an additional experiment was performed under oxidative stress conditions. HaCaT and HDF cells were exposed to 500 µM hydrogen peroxide (H_2_O_2_) for 24 h to induce cytotoxic stress. Exposure to H_2_O_2_ alone led to a decrease in cell viability of approximately 13%, confirming the effectiveness of this model in simulating oxidative damage. Co-treatment with Aloe gel and its fermented samples, applied at concentrations ranging from 5 to 25 mg/mL, significantly attenuated the deleterious effects of H_2_O_2_ and improved cell viability in both cell lines (Figure S4). These findings indicate that the tested preparations exhibited a genuine cytoprotective effect under stress conditions and that the increased metabolic activity observed in previous assays is not solely attributable to mitochondrial stimulation. Additionally, the Neutral Red assay performed on non-stressed cells further confirmed the beneficial effects of the tested samples, particularly in terms of preserving lysosomal integrity and membrane function. This may suggest that the aloe-based preparations supported basic cellular homeostasis even in the absence of external stress.

The positive effect of Aloe vera gel on keratinocytes and fibroblasts has already been shown by other authors who have studied the effect of this gel on the viability, proliferation and migration capacity of these cells [50,51]. Although there are studies available on the effect of fermented Aloe vera gel on skin cells, there are no literature reports on ferments obtained using kombucha. So far, studies conducted by other authors have focused on the assessment of the cytotoxicity of Aloe vera ferments obtained using Gluconacetobacter xylinus subsp. xylinus and Saccharomyces cerevisiae [13] or *Lactobacillus plantarum* [52,53,54].

The beneficial effects of fermented Aloe vera gel on skin cell viability are likely due to bioactive compounds produced during the fermentation process, which can increase mitochondrial activity and collagen production and inhibit the synthesis of matrix metalloproteinases. The cytoprotective effects may also be due to the ability of these phytochemicals to protect against oxidative stress and UV-induced damage [15,53,55]. The positive effect of the gel obtained by using kombucha–Aloe ferments on skin cells may be due to the action of aloesin because, as the literature data indicate, it can increase cell migration, support the release of cytokines and growth factors and also increase angiogenesis, which leads to accelerated wound closure [56]. Important compounds identified in the obtained samples that can affect skin cells are also aloin A and aloin B. These compounds can protect skin fibroblasts from oxidative damage caused by heat stress, accelerate wound healing by inducing the expression of epidermal growth factor and promote the formation of new blood vessels [57,58]. Phytochemicals present in the obtained gel and Aloe ferments that may have a positive effect on the viability, activity and proliferation of skin cells also include gallic acid, chlorogenic acids, rutoside, epigallocatechin, catechin, epicatechin and quercetin, and kaempferol derivatives. These compounds can increase the metabolic activity of skin cells, protect them from UV damage, reduce inflammation by affecting the levels of various cytokines and promote wound healing by stimulating cell migration [59,60,61,62]. The lack of cytotoxic effects of the obtained ferments on skin cells (in the appropriate concentration range) indicates that these bioferments can be perceived as a valuable cosmetic raw material with a multifaceted effect, which can positively influence the viability and appearance of skin cells.

### 2.5. Assessment of Extracellular Matrix (ECM) Degrading Enzymes Activity

Collagenase, elastase and hyaluronidase are key enzymes involved in the degradation of extracellular matrix components, contributing significantly to skin aging and the loss of structural integrity. Collagenase degrades collagen fibers, elastase degrades elastin and hyaluronidase depolymerizes hyaluronic acid, leading to a reduction in skin firmness, elasticity and hydration. Inhibiting the activity of these enzymes is a promising strategy for maintaining youthful skin appearance and function. Aloe vera gel, known for its anti-inflammatory, antioxidant and wound-healing properties, has been shown to have an inhibitory effect against these enzymes [63]. In addition, fermentation of Aloe vera by SCOBY increases the bioavailability of its active compounds, potentially enhancing its protective effect against enzymatic degradation of the skin matrix.

**Figure 7:**
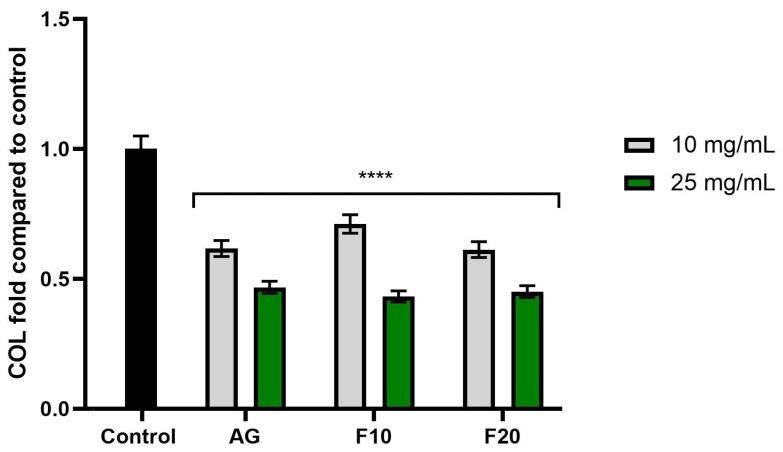
The impact of Aloe vera gel (AG) and its ferments (F10, F20) on the level of collagenase (COL) calculated as a percentage in comparison to the control. Data are mean ± SD from three independent experiments, in which each sample was tested in duplicate. **** *p* < 0.0001.

**Figure 8:**
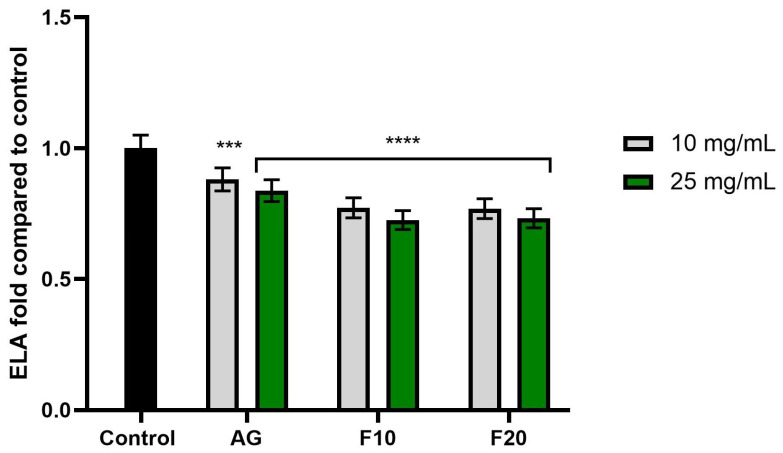
The impact of Aloe vera gel (AG) and its ferments (F10, F20) on the level of elastase (ELA) calculated as a percentage in comparison to the control. Data are mean ± SD from three independent experiments, in which each sample was tested in duplicate. **** *p* < 0.0001, *** *p* = 0.0062.

**Figure 9:**
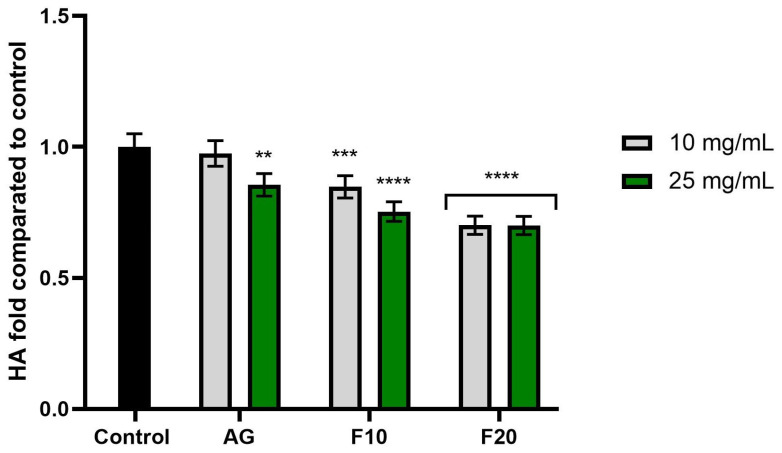
The impact of Aloe vera gel (AG) and its ferments (F10, F20) on the level of hyaluronidase (HA) calculated as a percentage in comparison to the control. Data are mean ± SD from three independent experiments, in which each sample was tested in duplicate. **** *p* < 0.0001, *** *p* = 0. 0.0015, ** *p* = 0.0009.

Figure 7, Figure 8 and Figure 9 show the results of collagenase, elastase and hyaluronidase activity. Values are expressed as the fold of the control (HDF cells not exposed to the tested compounds). As demonstrated in Figure 7, all tested substances at both tested concentrations exhibited a statistically significant inhibition of collagenase activity compared to the control. The higher concentration (25 mg/mL) showed a stronger inhibitory effect, reaching values of 0.432 ± 0.02, 0.451 ± 0.02 and 0.468 ± 0.02 fold for F10, F20 and AG, respectively.

In the case of elastase activity (Figure 8), no significant differences were observed between the tested concentrations. However, the most pronounced inhibitory effect was noted for the F10 and F20 ferments at a concentration of 25 mg/mL, reaching values of 0.725 ± 0.04 and 0.732 ± 0.04, respectively.

In turn, the analysis of hyaluronidase activity (Figure 9) showed statistically significant enzyme inhibition in the cases of F10 at a concentration of 25 mg/mL and F20 at both tested concentrations, the latter of which obtained values of 0.700 ± 0.04 (10 mg/mL) and 0.699 ± 0.03 (25 mg/mL) fold compared to the control, respectively.

As shown by studies and chromatographic analysis (Table 1), Aloe vera is a plant rich in bioactive compounds, including polyphenols, which are known to inhibit collagenase and elastase activity. The gel from the leaf pulp, rich in polysaccharides, including acemannan, is particularly effective in skin care. This effect is associated with the induction of collagen synthesis and the promotion of tissue cross-linking by stimulating cytokine production and macrophage activation [64]. Other studies have also shown that Aloe vera gel effectively inhibits excessive MMP expression induced by UV-B radiation, which was confirmed in a mouse model [65]. Furthermore, Chitra et al. suggested that the wound healing properties attributed to Aloe vera result from the increased collagen content in granulation tissue and its cross-linking [66].

The fermentation of plant extracts, including Aloe vera and *Aloe arborescens*, has been shown to cause an increase in their biological activity. A study published by Ro et al. showed that lactic acid fermentation with *Lactobacillus plantarum* significantly increased the antioxidant activity of Aloe extracts, improved collagen production and inhibited MMP-1 synthesis, indicating their potential use in anti-aging skin care products. Furthermore, their analysis of the bioferment composition showed a dominance of relatively low-molecular-weight polysaccharides (20% in the 600–900 Da) in contrast to unfermented Aloe gel, in which 95% of polysaccharides had a molecular weight in the 200,000–300,000 Da range. The lower molecular weight of polysaccharides is a result of the fermentation process, which improves their permeability through the skin barrier and potentially enhances the anti-aging effect [53].

Moreover, aloin A and B, present in Aloe vera gel and its kombucha ferments, have been recognized as effective inhibitors of *Clostridium histolyticum* collagenase (CFC) and matrix metalloproteinases (MMPs), including MMP-1 and MMP-3. The mechanism of action involves a reversible, non-competitive binding of aloin to the enzyme, which leads to the destabilization of its structure and the inhibition of enzymatic activity. The structural similarity of aloin to tetracyclines suggests a similar mechanism of inhibition, making it a potential natural inhibitor of MMPs [67]. Due to its ability to inhibit enzymes responsible for collagen and elastin degradation, aloin may contribute to delaying skin ageing processes and supporting its regeneration. Additionally, its antioxidant properties may protect skin cells from oxidative stress, which has been confirmed in studies on human skin fibroblasts [57,68,69].

### 2.6. Transepidermal Water Loss (TEWL) and Skin Hydration Measurements

Skin has a key protective function, preventing excessive water loss from the skin and acting as a barrier against the penetration of microorganisms and viruses. The physical skin barrier is formed by lipids and corneocytes of the *stratum corneum*, while lipids in combination with the secretions of the sebaceous glands co-form the biochemical barrier. The proper functioning of the skin barrier depends on the adequate hydration of the *stratum corneum*, the level of transepidermal water loss (TEWL) and the amount of sebum secreted. When the skin is damaged by chemical or physical agents, barrier function is impaired, as manifested by an increase in TEWL values [70]. In the present study, a tewameter was used to measure the amount of water vapor secreted from the skin, allowing a non-invasive assessment of the effectiveness of barrier function. In addition, the level of skin hydration after application of the gel and Aloe vera ferments was determined using a corneometer.

**Figure 10:**
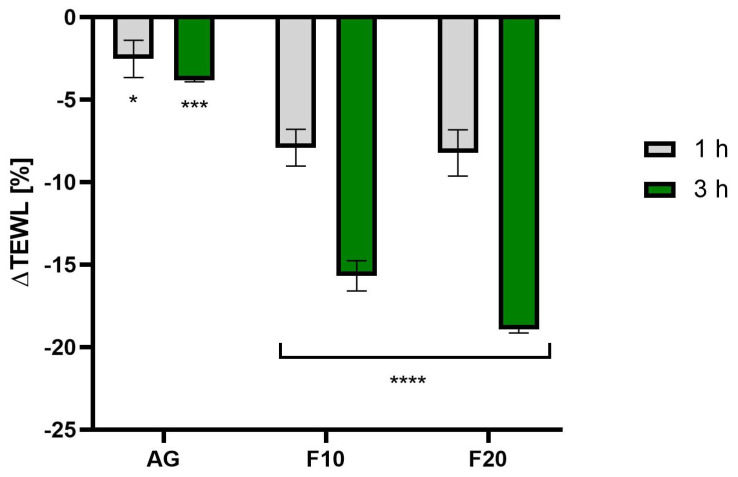
The impact of Aloe vera gel and its ferments (F10, F20) on transepidermal water loss (TEWL) expressed as a percentage compared to the control field. Data are the mean ± SD of three independent measurements. **** *p* < 0.0001, *** *p* = 0.0004, * *p* = 0.0255.

As shown in Figure 10, the most favorable changes in the reduction of TEWL values were observed after treatment with ferments. The values decreased, reaching 18.93 ± 0.21% and 15.68 ± 0.92% of the control field (untreated with the tested compound) for F20 and F10, respectively, 3 h after application. In addition, the time of application had a significant effect on the results—a comparison of the values after 1 and 3 h indicated that a longer duration of action led to more favorable results, especially for samples with ferments.

**Figure 11:**
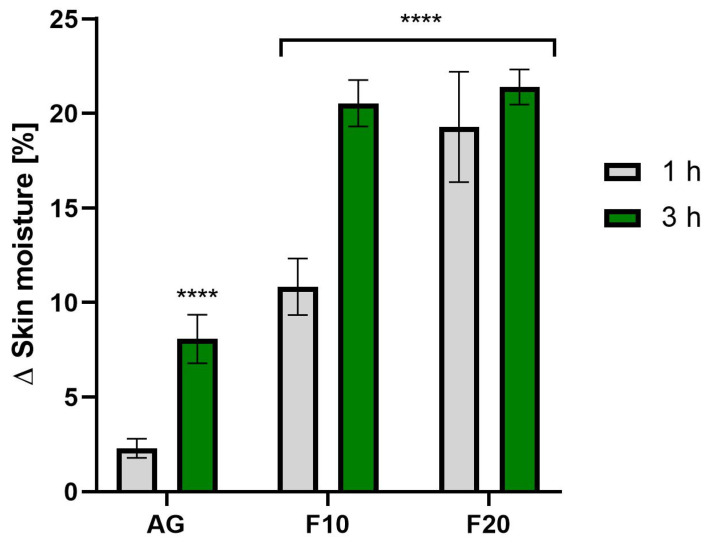
The impact of Aloe vera gel and its ferments (F10, F20) on skin moisture expressed as a percentage compared to the control field. Data are the mean ± SD of three independent measurements. **** *p* < 0.0001.

The analysis of the level of skin hydration (Figure 11) also confirmed the positive effects of Aloe vera gel and its kombucha ferments. It was observed that, in the case of F20, exposure time did not have as significant an effect on hydration levels as in the case of F10. The highest hydration level was observed for F20 at 3 h after application, reaching an increase of 21.40 ± 0.93% compared to the control field.

Skin hydration and transepidermal water loss are critical to maintaining barrier integrity. Aging damages dermal connective tissues, reducing elasticity, while UVA and UVB rays contribute to inflammation and melanin production, respectively. Although new topical treatments are emerging, many have raised safety concerns, highlighting the need for safer, more effective anti-aging products [71]. Fermentation technology improves skin care by increasing the bioavailability of both plant and bacterial ingredients. The process breaks down bioactive molecules found in plants, making them smaller and more easily absorbed by the skin [17]. Although there are no studies evaluating the level of skin hydration after kombucha fermentation, there are studies on products based on biofermented Aloe vera using *Lactobacillus* bacteria. Aloe vera gel is rich in hydrophilic compounds, including mono- and polysaccharides (especially acemannan and glucomannan) and amino acids, which act as humectants. These substances attract and retain water in the *stratum corneum*, increasing hydration [3]. Studies suggest that the fermentation process increases the bioavailability of polyphenols and polysaccharides contained in Aloe vera. As shown, fermented Aloe *L. plantarum* indicate that high-molecular-weight Aloe polysaccharides (~200–300 kDa) can be enzymatically degraded to low-molecular-weight oligosaccharides (~600–900 Da), increasing skin penetration and improving biological properties. At the same time, SCOBY bacteria and lactic acid bacteria contained in kombucha are known to synthesize microbial exopolysaccharides—mainly low molecular weight levans, which contribute to higher viscosity and can form moisturizing films on the skin surface [55,72]. These molecules help increase skin hydration by enhancing natural moisturizing factors (NMF) [15,47].

## 3. Materials and Methods

### 3.1. Plant Materials and Fermentation Procedure

Aloe leaves were collected from controlled organic plantations where no artificial fertilizers or pesticides were used. The starter culture of kombucha mushroom (SCOBY) was obtained from a commercial supplier in Poland. It was a symbiotic culture consisting mainly of acetic acid bacteria and yeast. The pulp of Aloe leaves was separated, then covered with water in a ratio of 1:1 and blended, obtaining an Aloe gel with a concentration of 1000 mg/mL. The preparation was sweetened with sucrose (10% (*w*/*v*)) and transferred to glass beakers (1000 mL; height 18 cm, diameter 8 cm) for fermentation. Then, the tea fungus SCOBY and the starter liquid of kombucha were added, reaching a final concentration of 10% (*v*/*v*). Fermentation was carried out at room temperature for 10 and 20 days. Ferments collected after 10 and 20 days, respectively, were designated as F10 and F20. Unfermented Aloe gel was designated as AG.

### 3.2. Determination of Biologically Active Compounds

The separation of analytes was carried out using an Infinity II Series ultra-high-performance liquid chromatography (UHPLC) system equipped with a diode array detector (DAD) and an Agilent 6224 ESI/TOF mass spectrometer (Agilent Technologies, Santa Clara, CA, USA). For chromatographic separation, a Kinetex C18 reversed-phase column (100 Å, 150 × 2.1 mm, 1.7 µm particle size; Phenomenex, Torrance, CA, USA) was employed. The chromatographic conditions followed a previously established method [73].

Spectral data were acquired over a wavelength range of 200–600 nm. The mass spectrometry settings included a drying gas temperature of 325 °C, a gas flow rate of 8 L/min, a nebulizer pressure of 30 psi, a capillary voltage of 3500 V, a fragmentor voltage of 220 V and a skimmer voltage of 65 V [74].

### 3.3. Ex Vivo Penetration

The permeation study was performed using Franz diffusion cells (SES GmbH Analyse Systeme, Bechenheim, Germany) with diffusion areas of 1 cm^2^. The diffusion cells were kept at a constant temperature of 37.0 ± 0.5 °C, which was maintained via a thermostat (VEB MLW Prüfgeräte-Werk type 3280, Leipzig, Germany). The volume of the acceptor chamber was 8 mL, and the volume of the donor chamber was 2 mL. The volume of the recipient chamber was 8 mL and was filled with PBS buffer (pH 7.4). The contents of the recipient chamber were mixed with a magnetic rod at the same speed in all cells. Porcine skin, whose properties and structure are similar to human skin, was used as a skin model [35]. The skin was supplied from a local slaughterhouse. The samples were wrapped in aluminum foil and stored in a freezer at −20 °C for no longer than three months, which did not negatively affect the barrier properties of the skin [75]. On the day of the experiment, the samples were slowly thawed at room temperature for 30 min [76,77,78]. Intact skin fragments (impedance-checked) were placed between the donor and recipient chambers. All diffusion units were then stabilized at 37 °C for 15 min. Impedance measurement using an LCR 4080 m (Voltcraft, Conrad Electronic, Hirschau, Germany) operating in parallel mode at a frequency of 120 Hz (measurement error at values in the kΩ range <0.5%) was used to verify skin integrity. The probe tips were placed in the donor and recipient chambers, which were filled with PBS buffer as previously described [79,80]. Only skin samples with impedance exceeding 3 kΩ, which corresponds to the values characteristic for human skin [81], were qualified for the experiment. A strictly defined amount (1 mL) of plant extracts was applied to the skin surface on the side of the donor chamber. The donor chambers were secured with plastic stoppers to limit evaporation of the solution. The penetration test lasted 24 h. Additionally, in a separate experiment, 1 g of each tested hydrogel was applied to the skin. Samples (0.3 mL) from the recipient chamber were taken after 1, 2, 3, 5, 8 and 24 h, and each time the collected volume was supplemented with fresh PBS buffer (pH 7.4). The concentration of active substances in the acceptor phase was determined using the HPLC method. On this basis, the cumulative mass (in µg) of penetrating compounds was calculated.

The accumulation of selected polyphenols in the skin after 24 h was assessed according to the modified method described by Ossowicz-Rupniewska et al. [82]. After the experiment, each skin sample was gently washed with PBS buffer (pH 7.4), then a fragment of 1 cm^2^ corresponding to the diffusion area was cut out and dried at room temperature. These fragments were minced, placed in 2 mL of methanol and incubated for 24 h at 4 °C. After incubation, the samples were homogenized for 3 min (homogenizer IKA^®^ T18 digital ULTRA TURRAX, Staufen, Germany) and then centrifuged at 3500 rpm for 5 min. The obtained supernatant was collected for further HPLC and spectrophotometric analyses; pure methanol was used as a control. The amount of accumulated phenolic acids in the skin was expressed as the mass of a given compound per skin mass (µg/g).

The identification of selected polyphenols in the acceptor fluid after permeation as well as the identification of fluid after skin extraction were carried out using an HPLC system from Knauer (Berlin, Germany) coupled with the WellChrom UV K-2600 detector. The tested components were separated on a 125 × 4 mm column containing Hypersil ODS C18 with a particle size of 5 µm. The detection of selected polyphenols was performed by UV absorption at λ = 278 nm. The compound was identified based on its retention time and by comparison with a standard under the same conditions. The mobile phase consisted of 1% aqueous acetic acid solution (A) and 100% MeOH (B). The samples were eluted with the following gradient: 90% A and 10% B from 0 to 6 min, 84% A and 16% B from 7 to 25 min, 72% A and 28% B from 26 to 37 min, 65% A and 35% B from 38 to 47 min, 50% A and 50% B from 48 to 64 min and 90% A and 10% B from 65 to 70 min in order to restore the initial conditions before the injection of a new sample. The flow rate was 0.8 mL/min, and the injection volume was 20 µL. All samples were analyzed three times [83].

### 3.4. Assessment of Antioxidant Activity

#### 3.4.1. DPPH Radical Scavenging Assay

The antioxidant activity of Aloe vera gel and its kombucha ferments was assessed using the DPPH (1,1-diphenyl-2-picrylhydrazyl) assay based on a modified published method [43]. A 4 mM DPPH methanolic solution was mixed with test samples at 5, 10, 25 and 50 mg/mL. Absorbance was recorded at 517 nm with a UV-VIS spectrophotometer (Thermo Fisher Scientific, Waltham, MA, USA). Water mixed with DPPH served as the control. Each concentration was tested in triplicate across three independent experiments. The results were expressed as the percentage of DPPH scavenging compared to the control.

(1) $$\%\;DPPH\;scavenging=\frac{Abs\;control-Abs\;sample}{Abs\;control}\times 100$$

#### 3.4.2. ABTS Radical Scavenging Assay

The antioxidant capacity of Aloe vera gel and its ferments was also assessed using the ABTS ^+^ assay following a modified published method [84]. A 7 mM ABTS ^+^ solution was mixed with 2.4 mM potassium persulfate and incubated in the dark for 16 h at room temperature. This mixture was then diluted with methanol to an absorbance of ~1.0 at 734 nm. Test samples (5, 10, 25 and 50 mg/mL) were added to the ABTS^+^ solution, and absorbance was measured at 734 nm with a UV-VIS spectrophotometer (Thermo Fisher Scientific, Waltham, MA, USA). Methanol with ABTS ^+^ served as the control. Each concentration was tested in triplicate across three independent experiments. The results were expressed as the percentage of ABTS ^+^ scavenging compared to the control.

(2) $$\%\;ABTS\;scavenging=\left(1-\left(\frac{Abs\;sample}{Abs\;control}\right)\right)\times 100$$

#### 3.4.3. Determination of Intracellular Levels of Reactive Oxygen Species (ROS)

The antioxidant activity of Aloe vera gel and its kombucha ferments was evaluated by measuring reactive oxygen species (ROS) levels in human fibroblasts (HDF) and keratinocytes (HaCaT) using the H2DCFDA fluorescent probe. Cells were cultured, treated with various concentrations (5–50 mg/mL) and then exposed to oxidative stress induced by 500 µM hydrogen peroxide. ROS levels were quantified via fluorescence measurement (λ_ex = 485 nm, λ_em = 530 nm) using a microplate reader (ThermoFisher Scientific, Waltham, MA, USA). The negative controls received no treatment, while the positive controls were treated with H_2_O_2_ only. All tests were conducted in triplicate across three independent experiments. [85].

### 3.5. Cell Culture and Cytotoxicity Assessment Using Alamar Blue and Neutral Red Assays

The cytotoxicity of the tested samples was assessed on normal human fibroblast cell lines (HDF) and keratinocyte lines (HaCaT), which were obtained from CLS Cell Lines Service (Eppelheim, Germany). Both cell types were cultured in DMEM medium (Dulbecco’s Modified Eagle’s Medium, Capricorn Scientific GmbH, Ebsdorfergrund, Germany). This medium additionally contained phenol red, sodium pyruvate, L-glutamine, high glucose (4.5 g/L), FBS (10% (*v*/*v*)) and antibiotics (1.0% (*v*/*v*), 100 U/mL penicillin and 1000 μg/mL streptomycin (Thermo Fisher Scientific, Waltham, MA, USA). The cells were cultured at 37 °C in a humidified atmosphere containing 5% CO_2_. To prepare cultures for analysis, both cell types were seeded separately on 96-well culture plates at a density of 1 × 10^4^ per well. Cells for the AB test were seeded on black flat-bottom plates, and cells for the NR test were seeded on transparent plates (Googlab Scientific, Rokocin, Poland). After an initial 24-h cell culture on plates, the cells were exposed to the tested gel and ferments in the concentration range of 0.5–5.0% by dissolving the tested samples in complete DMEM culture medium. The incubation time of cells with the tested samples for both tests was 24 h. The control cells were HaCaT and HDF cells separately cultured in complete DMEM medium without the addition of gel and Aloe ferments, for which the viability was assumed to be 100%.

The assessment of skin cell viability by measuring the reduction of water-soluble resazurin sodium salt (AB assay, Merck KGaA, Darmstadt, Germany) and by assessing the storage capacity of neutral red dye in lysosomes (NR assay, Merck KGaA, Darmstadt, Germany) was performed according to the previously described methodology [86]. In the case of the AB test, after the exposure of cells to individual concentrations of gel and ferments, the solution from the wells of the black plate was aspirated and replaced with 60 µM resazurin working solution. The cells prepared in this way were incubated with resazurin for 2 h. Then, fluorescence was measured at an excitation wavelength of 530 nm and an emission wavelength of 590 nm using a microplate reader (ThermoFisher Scientific, Waltham, MA, USA). In the case of analyses using neutral red, after 24 h of incubation, the medium with the tested samples was aspirated and replaced with a 40 µg/mL solution of this dye. The prepared samples were incubated for 2 h. In the next step, the cells were washed twice with phosphate-buffered saline (PBS, Genos, Łódź, Poland), a destaining buffer (C_2_H_5_OH/CH_3_COOH/H_2_O, 50.0%/1.0%/49.0%) was added to the wells and the plates were shaken for 15 min to release the dye accumulated in the cell lysosomes. Then, the absorbance of the samples was measured at λ = 540 nm using the above-mentioned microplate reader. The cytotoxicity analysis of the tested samples was carried out by performing three independent experiments, in which each concentration of the gel and ferments was tested three times.

### 3.6. Assessment of Extracellular Matrix (ECM) Degrading EnzymesActivity Using ELISA Method

In order to measure the ability of Aloe vera gel and its ferments, the enzymatic activity of collagenase, hyaluronidase and neutrophil elastase were investigated via spectrophotometric analyses performed using the human COL2 α 1 ELISA kit, human HAase ELISA kit and human NE/ELA2 ELISA kit (Elabscience Biotechnology Inc., Houston, TX, USA) according to the manufacturer’s instructions. Human fibroblasts were cultured, treated with the extract and ferments (at the concentrations of 10 and 25 mg/mL), lysed and subjected to ELISA protocols involving antibody incubation, washing, detection with biotinylated antibodies and HRP conjugates and the measurement of absorbance with a 450 nm microplate reader (ThermoFisher Scientific, Waltham, MA, USA). Results were expressed as fold compared to the control (HDF cells not treated with test compounds.

### 3.7. Transepidermal Water Loss (TEWL) and Skin Hydration Measurements

Transepidermal water loss (TEWL) and skin hydration were measured using a TEWAmeter TM 300 and Corneometer CM825, respectively, both connected to an MPA adapter (Courage + Khazaka Electronic, Köln, Germany). Ten volunteers participated in the study. All participants gave their informed consent for inclusion before they participated in the study. The scope of the study was in line with Regulation (EC) No 1223/2009 of the European Parliament and the Council, Cosmetics Europe–The Personal Care Association Guidelines “Product Test Guidelines for the Assessment of Human Skin Compatibility 1997”, Cosmetics Europe–The Personal Care Association’s “Guidelines for the Evaluation of the Efficacy of Cosmetic Products 2008” and the World Medical Association’s (WMA) Declaration of Helsinki’s Ethical Principles for Medical Research Involving Human Subjects. Three 2 × 2 cm areas were marked on each volunteer’s forearm; two were treated with 100 µL of the Aloe vera gel and kombucha ferments, while one remained untreated as a control. Measurements were taken after 1 and 3 h. Skin hydration was assessed based on the mean of 5 measurements, and TEWL was derived from 20 measurements per volunteer. Results were expressed as a percentage relative to the untreated control field [87].

### 3.8. Statistical Analysis

The data are presented as means ± SD of three independent experiments. The obtained experimental data were analyzed with one-way analysis of variance (ANOVA), followed by Dunnett’s and Tukey’s post-test. The statistical significance was determined at **** *p* < 0.0001, *** *p* < 0.001, ** *p* < 0.01 and * *p* < 0.05 compared with the control. The statistical analysis was performed using GraphPadPrism 8.4.3. (GraphPad Software, Inc., San Diego, CA, USA).

## 4. Conclusions

Aloe vera gel, due to its high content of polysaccharides, exhibits moisturizing properties and is widely used in cosmetic products. The purpose of this study was to evaluate the effects of the fermentation of Aloe vera gel with a tea fungus (kombucha) on the phytochemical composition and biological properties of the obtained products. The fermentation resulted in an increase in the content of biologically active compounds. The most abundant components were aloesin, aloin A and aloin B. Pig skin permeation analysis showed that the fermented samples contained active compounds such as gallic acid, catechin, chlorogenic acid and rutoside, which effectively accumulated in the skin. Antioxidant potential was also evaluated by DPPH, ABTS and intracellular assays on HaCaT and HDF cell lines. Ferments, especially after 20 days of fermentation, showed significantly higher antioxidant activity. Cytotoxicity tests confirmed the ferments’ lack of toxic effects on fibroblasts and keratinocytes, and, in some cases, a cytoprotective effect was noted. In addition, ELISA tests showed a significant inhibition of collagenase, elastase and hyaluronidase enzymes for both Aloe vera gel and its ferments (F10 and F20). The kombucha ferments also showed better moisturizing properties and lower transepidermal water loss (TEWL). This study confirms that Aloe vera gel fermentation using kombucha is an effective method for increasing the bioactivity of plant compounds, providing an alternative to fermentation with lactic acid bacteria.

## Figures and Tables

**Table 1:** Mass data and the results of quantitative analysis of Aloe vera gel (AG) and kombucha ferments performed using UHPLC/DAD/ESI-MS. The values (µg/mL) represent means ± standard deviation (SD) of triplicate.

R_T_ (min.)	Mass Data (*m*/*z*-H)	Component	AG	F10	F20
4.88	169.01502	Gallic acid	nd	6.46 ± 0.61	11.54 ± 0.88
6.24; 7.18	343.06757	Galloylquinic acids	nd	2.06 ± 0.18	2.75 ± 0.18
9.60	305.06768	Gallocatechin	nd	0.66 ± 0.03	0.87 ± 0.04
11.4; 16.6	353.08886	Chlorogenic acids	nd	2.01 ± 0.10	2.75 ± 0.07
14.47	305.06777	Epigallocatechin	nd	2.41 ± 0.14	3.25 ± 0.18
15.62	393.12011	Aloesin	215.89 ± 5.8	216 ± 8.7	249 ± 9.3
15.70	289.07122	Catechin	nd	1.86 ± 0.07	2.10 ± 0.14
16.29	395.13477	8-C-glucosyl-aloesol	13.72 ± 0.54	12.98 ± 0.69	15.12 ± 0.78
18.14	407.13402	7-O-methyl aloesin	13.76 ± 0.75	12.95 ± 0.98	14.57 ± 1.02
19.60	289.07157	Epicatechin	nd	11.55 ± 0.72	16.72 ± 0.24
20.47; 22.82	337.09315	*p*-coumaryl quinic acids	0.92 ± 0.05	1.36 ± 0.04	1.42 ± 0.03
24.98	563.14333	Unknown flavonoid	nd	1.07 ± 0.01	1.52 ± 0.09
25.76	625.14049	Unknown flavonoid	nd	0.66 ± 0.03	0.95 ± 0.03
26.41	479.08291	Unknown flavonoid	nd	0.71 ± 0.01	1.08 ± 0.03
26.69; 27.04	447.12966	7-hydroxy-8-O-methylaloins	11.06 ± 0.65	10.02 ± 0.87	10.72 ± 0.74
27.88; 28.67; 32.22	433.11449	Hydroxyaloins	13.41 ± 0.87	23.81 ± 1.01	37.48 ± 2.25
27.9; 30.14	771.20112	Quercetin derivatives	nd	5.51 ± 0.14	8.63 ± 0,10
28.43	593.15317	Kaempferol derivative	nd	1.11 ± 0.10	1.78 ± 0.03
31.24	577.15903	Apigenin derivative	nd	1.20 ± 0.03	2.20 ± 0.03
31.98; 35.6	755.2036	Kaempferol derivatives	nd	1.88 ± 0.02	3.39 ± 0.14
32.06	609.1469	Rutoside	nd	1.71 ± 0.03	2.86 ± 0.03
32.63	463.08716	Quercetin galactoside	nd	0.50 ± 0.02	0.66 ± 0.05
33.74	463.0887	Quercetin glucoside	nd	0.43 ± 0.03	0.69 ± 0.02
37.31	447.09303	Kaempferol hexoside	nd	det	det
39.46	447.09222	Kaempferol 3-O-glucoside	nd	det	det
42.10	417.12098	Aloin B	27.1 ± 2.01	43.11 ± 2.74	55.86 ± 2.01
43.74	431.13492	Homonataloin B	2.82 ± 0.10	2.37 ± 0.12	3.03 ± 0.14
44.59	417.12009	Aloin A	79.10 ± 3.59	122.1 ± 5.57	158.9 ± 6.47

nd—not detected; det—detected.

**Table 2:** The content of selected active compounds in acceptor fluid after 1, 3, 5, 8 and 24 h penetration of 10-day (F10) and 20-day ferment (F20). ni: not identified. Different letters indicate significant differences between the extracts; (n = 3); α = 0.05.

Component	Time (h)	F10	F20
		[μg/cm^2^]	
Gallic acid	1	ni	ni
	3	ni	ni
	5	1.98 ± 0.12 ^b^	0.72 ± 0.10 ^a^
	8	2.64 ± 0.50 ^b^	1.12 ± 0.06 ^a^
	24	14.42 ± 2.69 ^a^	14.0 5 ± 2.69 ^a^
Chlorogenic acids	1	ni	ni
	3	ni	ni
	5	ni	ni
	8	ni	2.30 ± 0.61
	24	3.24 ± 0.59 ^b^	2.94 ± 0.54 ^a^
Catechin	1	ni	ni
	3	ni	ni
	5	ni	1.16 ± 0.28
	8	2.17 ± 0.36 ^b^	1.24 ± 0.28 ^a^
	24	9.41 ± 1.94 ^b^	6.32 ± 1.03 ^a^
Rutoside	1	ni	ni
	3	ni	ni
	5	5.48 ± 0.21	ni
	8	5.85 ± 0.13	ni
	24	6.61 ± 0.04 ^b^	4.49 ± 0.22 ^b^

## Data Availability

The data presented in this study are available on request from the corresponding author.

## Supplementary Materials

The following supporting information can be downloaded at: https://www.mdpi.com/article/10.3390/molecules30153192/s1, Figure S1: Base peak chromatograms in negative (red) and positive (blue) of Aloe extract; Figure S2: UV-Vis and MS spectra of the main constituents identified as aloin (upper panel) and aloesin (lower panel); Table S1: MS data compounds identified in the aloe water extract [88,89,90,91,92,93,94].
