# Supplementary material for: Enhancing the Cosmetic Potential of Aloe Vera Gel by Kombucha-Mediated Fermentation: Phytochemical Analysis and Evaluation of Antioxidant, Anti-Aging and Moisturizing Properties
Source: Molecules. 2025 Jul 30;30(15):3192. doi: 10.3390/molecules30153192 (PMC12348975; doi:10.3390/molecules30153192)
Supplement: Supplementary file 1 [file molecules-30-03192-s001.zip › molecules-3726438-supplementary.pdf]

molecules

MDPI

Article

Enhancing the Cosmetic Potential of Aloe Vera Gel by Kombucha-Mediated Fermentation: Phytochemical Analysis and Evaluation of Antioxidant, Anti-Aging and Moisturizing Properties

Aleksandra Ziemlewska [1], Martyna Zagórska-Dziok [1], Anna Nowak [2], Anna Muzykiewicz-Szymańska [2], Magdalena Wójciak [3], Ireneusz Sowa [3], Dariusz Szczepanek [4] and Zofia Nizioł-Łukaszewska [1,*]

[1] Department of Technology of Cosmetic and Pharmaceutical Products, Medical College, University of Information Technology and Management in Rzeszow, Sucharskiego 2, 35-225 Rzeszow, Poland; aziemlewska@wsiz.edu.pl (A.Z.); mzagorska@wsiz.edu.pl (M.Z.-D.)
[2] Department of Cosmetic and Pharmaceutical Chemistry, Pomeranian Medical University in Szczecin, 72 Powstańców Wielkopolskich Street, 70-111 Szczecin, Poland; anna.nowak@pum.edu.pl (A.N.); anna.muzykiewicz@pum.edu.pl (A.M.-S.)
[3] Department of Analytical Chemistry, Medical University of Lublin, Aleje Raclawickie 1, 20-059 Lublin, Poland; magdalena.wojciak@umlub.pl (M.W.); ireneusz.sowa@umlub.pl (I.S.)
[4] Department of Neurosurgery and Paediatric Neurosurgery, Medical University of Lublin, 20-090 Lublin, Poland; dariusz.szczepanek@umlub.pl
* Correspondence: zniziol@wsiz.edu.pl

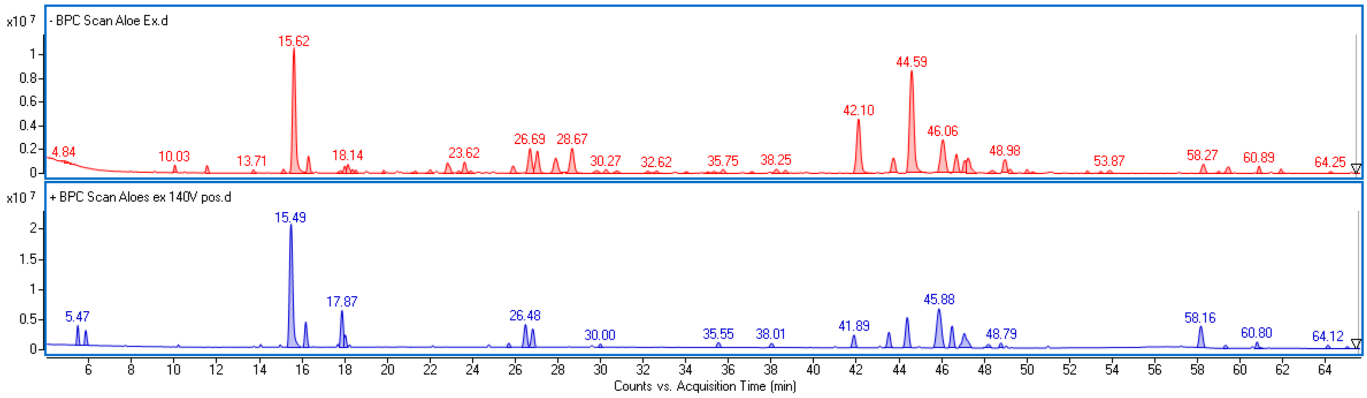

Figure S1. Base peak chromatograms in negative (red) and positive (blue) of Aloe extract.

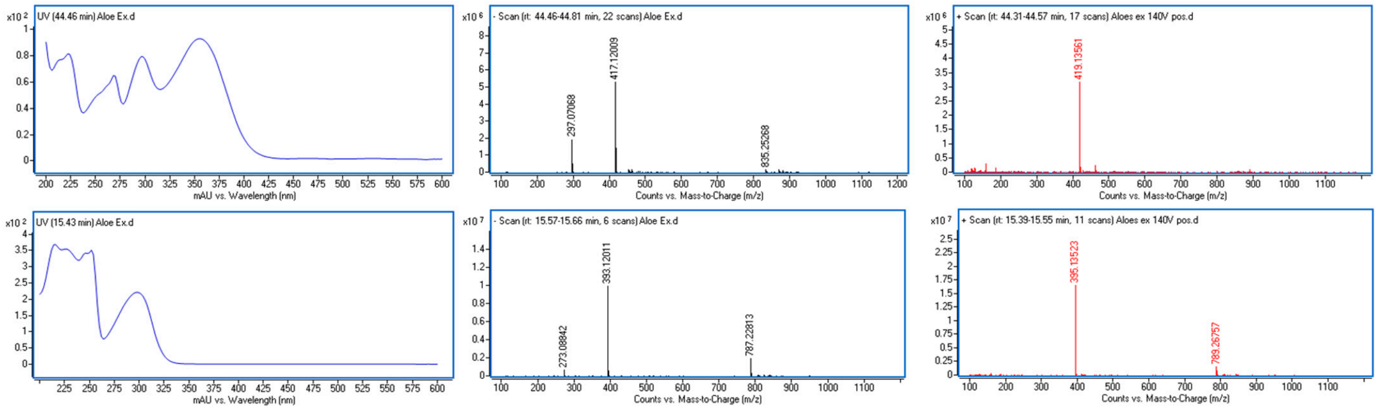

Figure S2. UV-Vis and MS spectra of the main constituents identified as aloin (upper panel) and aloesin (lower panel).

Table S1. MS data compounds identified in the aloe water extract.

R_T (min.)	Mass data [m/z-H]/[m/z+H]+	Formula	Δ ppm	Compound	Ref.
15.62	393.12011 (273, 245)/ 395.13523	$C_{19}H_{22}O_9$	2.55 3.99	Aloesin	[88–90]
16.29	395.13477 397.14989	$C_{19}H_{24}O_9$	0.04 1.47	8-C-glucosyl-aloesol	[89–91]
18.14	407.13402 409.14983	$C_{20}H_{24}O_9$	-1.85 1.28	7-O-methyl aloesin	[89]
18.39	409.15052 411.16501	$C_{20}H_{26}O_9$	0.28 0.12	8-C-glucosyl-7-methoxy-aloesol	[89–91]
20.47	337.09315 (191)	$C_{16}H_{18}O_8$	0.77	p-coumaryl quinic acid	[92,93]
22.82	337.09315 (191)	$C_{16}H_{18}O_8$	0.77	p-coumaryl quinic acid	[92,93]
23.62	527.10387	$C_{22}H_{24}O_{15}$	-0.71	Aloin derivative	
26.69	447.12966 (327) 449.14521	$C_{22}H_{24}O_{10}$	-0.02 2.20	7-hydroxy-8-O-methylaloin	[88,89]
27.04	447.13001 (327) 449.14538	$C_{22}H_{24}O_{10}$	0.76 2.58	7-hydroxy-8-O-methylaloin	[88,89]
27.88	433.11449 (270)/-	$C_{21}H_{22}O_{10}$	1.08	Hydroxyaloin	[88,90]
28.67	433.11468 (270)/-	$C_{21}H_{22}O_{10}$	1.52	Hydroxyaloin	[88,90]
32.22	433.11498 (270)/-	$C_{21}H_{22}O_{10}$	2.21	Hydroxyaloin	[88,90]
38.25	539.15601 (375)/ 541.17086	$C_{28}H_{28}O_{11}$	0.23 0.78	Aloeresin A	[88,89]
42.10	417.12098 (297)/ 419.13422	$C_{21}H_{22}O_9$	4.48 1.34	Aloin B	[88,89]
43.74	431.13492 (311, 297)/ 433.14978	$C_{22}H_{24}O_9$	0.38 1.09	Homonataloin B	[88,89]
44.59	417.12009 (297)/ 419.13561	$C_{21}H_{22}O_9$	2.35 4.67	Aloin A	[88,89]
46.06	555.18792/ 557.20194	$C_{29}H_{32}O_{11}$	1.32 0.36	isoaloeresin D	[88,89]
46.69	431.13505 (311,297)/ 433.5022	$C_{22}H_{24}O_9$	0.68 2.11	Homonataloin A	[88,89]
47.09	459.12992 (297)	$C_{23}H_{24}O_{10}$	0.54	6-O-acetyl-aloin B	[88,94]
47.24	553.17201/ 555.18659	$C_{29}H_{30}O_{11}$	0.86 0.91	7-O-methylaloeresin A	[88]
48.98	459.13025 (297)	$C_{23}H_{24}O_{10}$	1.26	6-O-acetyl-aloin A	[88,94]
58.16	539.19306/ 541.20702	$C_{29}H_{32}O_{10}$	1.46 0.36	Aloeresin	[88,89]
64.25	269.04605/ 271.06101	$C_{15}H_{10}O_5$	1.86 3.37	Aloe-emodin	[90]
